# Repurposing metformin for cancer treatment: current clinical studies

**DOI:** 10.18632/oncotarget.8194

**Published:** 2016-03-19

**Authors:** Young Kwang Chae, Ayush Arya, Mary-Kate Malecek, Daniel Sanghoon Shin, Benedito Carneiro, Sunandana Chandra, Jason Kaplan, Aparna Kalyan, Jessica K. Altman, Leonidas Platanias, Francis Giles

**Affiliations:** ^1^ Northwestern Medicine Developmental Therapeutics Institute, Chicago, IL, USA; ^2^ Robert H. Lurie Comprehensive Cancer Center of Northwestern University, Chicago, IL, USA; ^3^ Northwestern University Feinberg School of Medicine, Chicago, IL, USA; ^4^ David Geffen School of Medicine, University of California Los Angeles, Los Angeles, CA, USA; ^5^ Division of Hematology-Oncology, Department of Medicine, Jesse Brown VA Medical Center, Chicago, IL, USA

**Keywords:** metformin, clinical trials, cancer

## Abstract

In recent years, several studies have presented evidence suggesting a potential role for metformin in anti-cancer therapy. Preclinical studies have demonstrated several anticancer molecular mechanisms of metformin including mTOR inhibition, cytotoxic effects, and immunomodulation. Epidemiologic data have demonstrated decreased cancer incidence and mortality in patients taking metformin. Several clinical trials, focused on evaluation of metformin as an anti-cancer agent are presently underway. Data published from a small number of completed trials has put forth intriguing results. Clinical trials in pre-surgical endometrial cancer patients exhibited a significant decrease in Ki67 with metformin monotherapy. Another interesting observation was made in patients with breast cancer, wherein a trend towards improvement in cancer proliferation markers was noted in patients without insulin resistance. Data on survival outcomes with the use of metformin as an anti-cancer agent is awaited. This manuscript will critically review the role of metformin as a potential cancer treatment.

## INTRODUCTION

Metformin is one of the most widely prescribed oral anti-diabetic medications. It is the first line therapy for type 2 diabetes mellitus [1]. It has an anti-hyperglycemic effect which is mediated by inhibiting gluconeogenesis, decreasing glucose absorption from the small intestine, increasing glucose uptake in cells, and decreasing plasma free fatty acid concentration [2]. Metformin also increases insulin induced translocation of glucose transporters to the cellular plasma membrane, thus reducing insulin resistance [3]. Use of metformin has been found to be generally safe, with mild gastrointestinal symptoms being the most common adverse effects [4].

There is substantial preclinical evidence suggesting that metformin has anti-cancer properties. *In*-vitro and *in*-vivo analysis of metformin has exhibited anti-proliferative activity by inhibiting intracellular pathways. It has also been observed that metformin activates the T cell mediated immune response against cancer cells.

Numerous retrospective studies have reported that metformin is associated with a reduced risk of developing cancer. Meta-analysis of data obtained from cohort and observational studies has revealed that metformin use was associated with a decrease in both cancer related and all-cause mortality.

Here we summarize the available evidence from clinical trials of metformin as part of cancer therapy. We review the landscape of current investigation and suggest directions for future investigation.

## PRECLINICAL DATA

Metformin has been extensively studied in preclinical models, which has revealed numerous molecular pathways that it modulates, either directly or through other downstream targets, contributing to reduction in growth and proliferation of tumor cells. The inhibition of mTOR (mammalian target of rapamycin) in tumor cells is one of the potential key mechanisms that facilitates the anti-cancer activity of metformin. Use of metformin in MCF-7 breast cancer cells exhibited reduction in phosphorylation of S6 kinase, ribosomal protein S6 and eIF4E binding protein, along with inhibition of mTOR and reduced translation initiation due to AMPK activation [5]. Animal models of pancreatic cancer fed with metformin showed inhibition of insulin like growth factor-1 (IGF-1) and mTOR, along with an increase in phosphorylated AMPK and tuberous sclerosis complex (TSC1, TSC2) [6]. The AMPK mediated phosphorylation of TSC2 has been observed to increase the activity of TSC2, leading to inactivation of mTOR [7, 8]. AMPK has been described to directly inhibit mTORC1 through phosphorylation of mTOR binding raptor as well [9]. Comparing the effects of metformin with rapamycin, a direct mTOR inhibitor, metformin decreases the activation of AKT in addition to AMPK dependent mTOR inhibition. Thus, metformin renders a better anti-tumor response than rapamycin in breast cancer cells [10]. Metformin has been found to decrease HER2 expression in human breast cancer cells by directly inhibiting p70S6K1, which is a downstream effector of mTOR [11]. In a study using nude mice with acute myeloid leukemia (AML), the use of metformin was correlated with a decrease in proliferation of AML cells. This action was characterized by the activation of LKB1/AMPK/TSC pathway, which led to mTOR inhibition and consequently suppression of mRNA translation [12]. In tobacco carcinogen induced lung cancer mice, the inhibition of insulin like growth factor 1 receptor/insulin receptor (IGF- 1R/IR) by metformin decreased the downstream signaling through Akt pathway. This reduced the activation of mTOR in lung tissue which corresponded to a 72% reduction in tumor burden [13].

Metformin can inhibit the activation of mTOR independent of AMPK pathway as well. Metformin has been shown to escalate the expression of REDD1 by a p53 mediated inhibition of mTOR in prostate cancer cells [14]. Another study described that metformin can prevent mTOR activation through Ras-related GTPase (RagGTPases), independent of AMPK, as well as TSC1/2 [15].

Tumors are known to exhibit the Warburg effect, where tumor cells generate ATP from glycolysis instead of oxidative phosphorylation secondary to low nutrient supply and hypoxia [16]. Metformin blunts the Warburg effect and consequently downregulates the growth of cancer stem cells [17]. *In-vivo* studies on hepatocellular carcinoma xenografts have shown that metformin improves cellular oxygenation ability and decreases mitochondrial oxygen consumption, thus suppressing hypoxia-induced HIF-1α accumulation. These effects form the basis of anti-cancer activity of metformin, particularly against hepatocellular carcinoma [18].

In addition to the above, various other mechanisms of metformin for cell growth inhibition have been identified in pre-clinical models. Metformin therapy in paired isogenic colon cancer cell lines (HCT116 p53 [+/+] and HCT116 p53 [−/−]) showed an increase in apoptosis of p53 deficient cells [19]. In-vitro and in-vivo analysis of metformin therapy showed that it can inhibit growth of ovarian stem cells [20], glioma initiating cells [21], breast cancer cells [22], endometrial cancer cells [23] and non-small cell lung cancer cells [24]. It has also exhibited synergistic action with VEGF inhibitors to inhibit proliferation of BRAF mutant melanoma cells [25]. Additionally, it has been noted that metformin increases radiosensitivity of cancer cells [22, 24].

**Figure 1:**
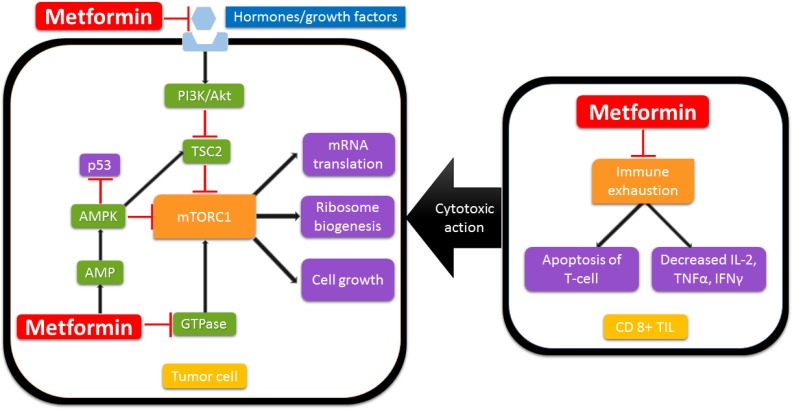
Possible mechanisms of anti-cancer activity of metformin The inhibition of mTORC1 is mediated by reducing the downstream effects of IGF-1 and insulin hormone receptor binding, increased TSC2 phosphorylation by AMPK, Rag GTPase mediated inhibition and phosphorylation of mTOR binding raptor by AMPK. This leads to inhibition of mRNA translation, ribosomal biogenesis and cell growth. Metformin also potentiates the immune system by reducing immune exhaustion of CD 8+ tumor infiltrating lymphocytes through phenotypic switching to memory cells, thus enhancing the immune response to cancer cells. Abbreviations: AMP, Adenosine monophosphate; AMPK, AMP activated protein kinase; GTPase, Ras-related GTPase; mTORC1, Mammalian receptor of rapamycin; TSC2, Tuberous sclerosis complex protein 2; PI3K, Phosphoinositide 3 kinase; Akt, Protein kinase B; IL-2, Interleukin-2; TNFα, Tumor necrosis factor -α; IFNγ, Interferon γ

Recent experiments on animal models have also suggested that metformin has immune modulatory properties. Metformin inhibits immune exhaustion of CD8+ tumor induced lymphocytes (TIL), thereby enhancing T cell mediated immune response to tumor tissue. It decreases apoptosis of CD8+ tumor infiltrating lymphocytes (TILs), and also shifts the phenotype of CD8+ TILs expressing exhaustion markers (especially PD1 negative Tim3 positive) from central memory T cells (TCM, inactive against tumor cells) to effector memory T cells (TEM, active against tumor cells). The increase in TEM cell population has been found to correlate with regression of tumor cells [26]. In a study evaluating an experimental cancer vaccine, administration of metformin after vaccination in animal models showed an increase in CD8+ memory T cells which conferred protective immunity upon subsequent tumor challenge [27]. Figure 1 summarizes the effects of metformin on various cellular pathways.

## EPIDEMIOLOGY

There are numerous epidemiological studies that have put forth evidence suggesting utility of metformin as an anti-cancer agent.

Several observational and cohort studies have been conducted to assess the influence of metformin on cancer. Survival analysis in a cohort study on diabetic patients comparing metformin users (*n* = 4,085) to non-users (*n* = 4,085) reported a reduced risk of cancer (hazard ratio, HR = 0.63; 7.3% diagnosed with cancer in metformin users *versus* 11.6% in non-users) [28]. Meta-analysis performed on 37 studies, with a total of 1,535,636 patients comparing metformin users and non-users reported overall cancer incidence summary relative risk (SRR) as 0.73. The results also noted a reduction of cancer incidence for liver (78%), breast (6%), pancreatic (46%) and colorectal cancer (23%). However, no significant correlation could be derived between the use of metformin and incidence of prostate cancer [29]. Meta-analysis of 6 case control studies comparing 39,787 participants that were on metformin to 177,752 participants that were not, exhibited a lower risk of developing lung cancer in the metformin group (odds ratio, OR = 0.55; *p* < 0.001) [30]. Likewise, another meta-analysis showed a reduction in incidence of prostate cancer (RR = 0.88; *p* = 0.03) in patients who were on metformin treatment [31]. Meta-analysis of 35 observational studies reported a considerable correlation with using metformin to reduce the risk of developing all-cancer (OR = 0.73), liver cancer (OR = 0.34), colorectal cancer (OR = 0.83), pancreatic cancer (OR = 0.56), gastric cancer (OR = 0.83), and esophageal cancer (OR = 0.90) [32]. A recent meta-analysis of 265 studies showed the use of metformin or thiazolidinediones was associated with a lower incidence of cancer (RR = 0.86 and 0.93 respectively). But interestingly, insulin, sulfonylureas, and alpha glucosidase inhibitor use was associated with an increased incidence of cancer (RR = 1.21, 1.20, 1.10 respectively) [33].

Meta-analysis of retrospective studies has provided substantial evidence associating the use of metformin with a decrease in cancer related mortality. A cohort study, investigating mortality due to cancer in type 2 diabetes patients, showed a decrease in cancer related mortality risk (HR = 0.56) with the use of metformin [34]. Data from meta-analysis of 6 observational studies noted a significant correlation between using metformin and risk of cancer related mortality (OR = 0.65) [32]. Retrospective analysis of data from over 350 primary care practices in the United Kingdom found a decrease in cancer related mortality (HR = 0.85) in diabetic patients who were on metformin monotherapy when compared with those on other drugs for diabetes [35]. Another meta-analysis showed that the use of metformin in diabetic patients diagnosed with cancer was associated with a decrease in risk of all-cause mortality in cancers of breast (pooled relative risk, RR = 0.70; *p* = 0.003), ovary (RR = 0.44; *p* < 0.001), endometrium (RR = 0.49; *p* = 0.001) and colorectal cancer (RR = 0.70; *p* < 0.001) [36].

In light of the available preclinical and retrospective data suggesting anti-cancer properties of metformin, clinical trials are necessary to further investigate its role in cancer therapy.

## METHODS

Information about the study drug was obtained from clinicaltrials.gov (a service of United States National Institutes of Health), using the search query “metformin” and “cancer”. Of the 223 results obtained from the search engine, relevant drug trials were selected. The published clinical trials were obtained from PubMed using the same search query and choosing the “clinical trial” filter. The publications that focused on use of metformin for treatment of cancer in human subjects through a prospective clinical trial were identified and selected. The results of completed clinical trials were obtained from PubMed and online abstract library for professional societies such as the American Association of Cancer Research (AACR) and American Society of Clinical Oncology (ASCO). The AACR and ASCO online libraries were explored using the NCT number associated with the clinical trial. The authors, enrollment number, primary & secondary outcomes and primary location of conducting the research mentioned in abstracts collected from ASCO and AACR database were matched with the information obtained from clinicaltrials.gov to avoid any discrepancies in associating the abstracts to the appropriate trial.

## CLINICAL TRIALS

Presently, there are 55 ongoing clinical trials in various stages that are evaluating metformin as a monotherapy (11 trials, 20% of all ongoing trials using metformin as an anti-cancer agent) or in combination with cytotoxic chemotherapy (38 trials, 69%) and/or radiotherapy (6 trials, 11%) for the treatment of cancer (Tables 1, 2, 3, 4). These trials primarily focus on establishing the effects of metformin on markers of cellular proliferation, pathological response rate, progression free survival, and recurrence free survival. Also, certain trials are directed towards determining the maximum tolerable dose of metformin in specific tumors.

**Table 1:** Proof of concept for anti-tumor activity of metformin

Cancer type	Phase	Primary outcome/Conclusion	Dosing regimen	Combination	Enrollment No.	Status	Results	Clinical trial identifier/ Reference
Bladder	Phase II	Changes in Ki67 when comparing TURBT and cystectomy tissue.	Metformin 850mg and 20 mg of Simvastatin daily from the time of enrollment in study untill the day before surgery (an approximate duration of 12 weeks)	metformin, simvastatin	44	Not yet recruiting	NA	NCT02360618
Breast	Phase 0	Tumor growth assessed by changes in ln Ki67	Metformin + atorvastatin PO	metformin, atorvastatin	40	Recruiting	NA	NCT01980823
Breast	Phase II	Effects of metformin on AMPK/mTOR pathway	Metformin 1500mg/day, 500mg (AM) and 1000mg (PM) for at least 2 weeks prior to surgery	metformin monotherapy	35	Published	- Non-significant difference in decrease of ln (Ki67) for both invasive and DCIS breast cancers (p= 0.98) or invasive breast cancer alone (p = 0.43). - No significant variation noted in changes of ln (Ki67) when metformin arm compared to control arm (p = .47).	NCT00930579[41]
Breast	Phase II	Effects of metformin in phosphorylation of S6K, 4E-BP-1 and AMPK	Extended release metformin 1500mg QD for 14-21 days	metformin monotherapy	41	Completed	NA	NCT01266486
Breast	Phase II RCT	Primary endpoint is change in Ki67. This study estimates apoptosis by TUNEL by comparing core and corresponding surgical biopsies.	Metformin 850mg QD for 3 days followed by 850mg BID days 4-28	metformin monotherapy	200	Published	TUNEL levels elevated at surgery as compared to baseline biopsy (P<0.0001), no major difference between metformin arm and placebo arm; Ki67 labelling index and TUNEL levels at baseline & surgery in direct correlation; TUNEL levels higher with metformin than placebo in women without insulin resistance (metformin: +4%, IQR: 2–14, placebo: +2%, IQR: 0–7), while the inverse observed in those who had insulin resistance (metformin: +2%, IQR: 0–6, placebo +5%, IQR: 0–15).	EudraCT 2008-004912-10, Trial number S425/408 [42]
Breast	Phase II, single arm	Effect of metformin on tumor growth rate over 2 to 3 weeks	Metformin 500 mg PO TID for 2-3 weeks before surgery	metformin monotherapy	39	Completed	HOMA levels significantly reduced; insulin, leptin and CRP had non-significant reductions; TUNEL staining increased from 0.56 to 1.05, p= 0.004; Ki67 staining decreased from 36.5 to 33.5%, p= 0.016.	NCT00897884 [37]
Breast	Phase II RCT	Changes in Ki-67	Metformin 850-mg QD PO days 1- 3 for allowing time to adjust to GI symptoms; two 850mg tablets after dinner on days 4 - 28.	metformin monotherapy	200	Completed	No statistically significant changes in Ki67 with metformin, noting a mean proportional increase of 4.0% (95% CI: −5.6% to 14.4%) in 4 weeks; Women with HOMA ≤ 2.8: non-significant increase of Ki67 by 11.1% (95% CI: −0.6% to 24.2%); women with HOMA ≤ 2.8: non-significant mean proportional decrease in Ki67 by 10.5% (95% CI: −26.1% to 8.4%).	ISRCTN16493703 [40]
Breast	NA, RCT	To determine the anti-cancer mechanism of metformin in breast cancer	Metformin 500mg QD for 1 week followed by 1000mg BID until the day of surgery	metformin monotherapy	47	Published	Metformin therapy showed no effect on tumor size. With using Allred scoring (0-8)- - Mean change in pAMPK score: Metformin- 5.18 to 6.45 Control arm- 5.64 to 6.00 - Change in pAKT staining score: Metformin- 5.91 to 5.00 Control arm- 4.91 to 5.18 - Mean change in Ki67 staining: Metformin- decrease by 3.4% Control arm- stable - Caspase-3 staining: Metformin- reduced by 0.29% Control arm- no significant change	[39]
Breast, lung, liver, kidney, lymphoma	Phase I	Effect of metformin plus sirolimus compared to sirolimus monotherapy on p70S6K	Sirolimus 3mg daily alone for the first 7 days. From day 8-21, metformin 500mg QD. If tolerated, dose increased to 1000 mg QD on day 15. Patients developing grade 2 toxicity due to metformin to be maintained on 500mg QD for rest of study, while those with >grade 2 toxicity will be taken off study. Day 22 onwards, all will be on combination of sirolimus + metformin	metformin, sirolimus	64	Recruiting	NA	NCT02145559
Endometrial	Phase 0	Changes in Ki-67	Metformin 850mg PO QD	metformin monotherapy	16	Completed	65% patients showed a fall in Ki-67 staining with metformin; those who showed response to metformin had higher pre-treatment Ki67 levels; pAMPK, pS6, pAKT, p-4E-BP-1 and ER expression were reduced; PR unaffected; tumor proliferation decreased by 11.75% with metformin.	NCT01911247 [43]
Endometrial	Phase II	Changes in pS6	Metformin 500mg PO TID from enrollment in study till surgical staging. If side effects persist, dose will be reduced to 500mg PO BID.	metformin monotherapy	80	Not yet recruiting	NA	NCT02042495
Endometrial	Phase II RCT	Changes in Ki67, phosphorylated histone H3, estrogen receptor, progesterone receptor, and telomerase.	NA	metformin monotherapy	40	Recruiting	NA	NCT01877564
Endometrial	NA	Effect of metformin on tumor proliferation	Metformin 500mg TID from diagnostic biopsy to surgery (average of 36.6 days)	metformin monotherapy	11	Published	Metformin use decreased IGF-1, mean plasma insulin, IGFBP-7, Ki67 (9.7%, p= 0.02) and pS6 (31%, p= 0.03) staining; Ki67 and pS6 in control group similar in baseline biopsy and surgical specimens.	[44]
Endometrial	NA	Increase in s6K	Metformin 850mg QD for at least 7 days and up to 30 days before surgery	metformin monotherapy	30	Active, but not recruiting	NA	NCT01205672
Endometrial	NA	Effect of anti-diabetic dose of metformin on tumor proliferation	Metformin 1500 - 2250mg/day for 4-6 weeks before surgery	metformin monotherapy	31	Published	Ki-67 mean proportional decrease= 44.2%; topoisomerase IIα mean proportional decrease= 36.4%; PRP S6 and ERK1/2 levels showed significant reduction while pAMPK and p27 had significant elevation.	[45]
Head and Neck squamous cell	Phase 0	Changes in TOMM20 and MCT4 in tumor tissue	NA	metformin monotherapy	40	Recruiting	NA	NCT02083692
Lung	Phase II	Effect of metformin on plasma IL-6 levels	Treatment with either cisplatin 75mg/m^2^ + pemetrexed 500mg/m^2^ on day1 for 4 cycles or targeted therapy with gefitinib 250mg/day until disease progression. If plasma IL-6 >2.0 pg/mL after 2 cycles of chemotherapy/targeted therapy, metformin 500mg PO QD will be given on day 1 of cycle 3. If tolerated, metformin will be titrated to 500mg BID in next week (Cycle 3 day8) and 500mg TID in the 3^rd^ week (Cycle 3 day15); those receiving chemo will be given metformin for 12 weeks and those on targeted therapy will receive it till disease progression.	metformin, cisplatin, pemetrexed, gefitinib	48	Recruiting	NA	NCT01997775
Prostate	Phase II RCT	Changes in Ki67 staining using IHC	Extended release metformin PO QD for 4-12 weeks	metformin monotherapy	21	Active but not recruiting	NA	NCT01433913
Prostate	NA	Assess safety, tolerability and change in Ki67 and AMPK pathway associated markers with the use of neoadjuvant metformin	Metformin 500mg TID	Metformin monotherapy	24	Published	Metformin decreased Ki67 index by 29.5% per patient and 28.6 % per tumor; P-4EBP1 staining showed reduction and P-AMPK recorded no change; 3 cases developed grade 3 toxicity.	[48]

Abbreviations: PFS, progression free survival; RCT, Randomized control trial; TUNEL, Terminal deoxynucleotidyl transferase dUTP nick end labeling; P-AMPK, phosphorylated AMPK; HOMA, Homeostasis model assessment; IGF-1, insulin like growth factor-1; IQR, Interquartile ratio; PO, taken by mouth; QD, once a day; BID, two times a day; TID, three times a day

**Table 2:** Anti-tumor activity of metformin in locally advanced and hematologic cancers

Cancer type	Phase	Primary outcome/Conclusion	Dosing regimen	Combination	Enrollment No.	Status	Results	Clinical trial identifier/Reference
AML	Phase I	MTD of metformin plus cytarabine	Metformin PO BID on days 1-15 and cytarabine IV over 3 hours BID on days 4-10.	metformin, cytarabine	28	Recruiting	NA	NCT01849276
Breast	Phase II RCT	pCR rate (evaluated in 5 months)	Docetaxel 75mg/m^2^ IV, epirubicin 75mg/m^2^ IV and cyclophosphamide 500mg/m^2^ IV day 1 of each 21 day cycle; 6 cycles; metformin 500mg TID PO (500mg daily in 1st cycle)	metformin, docetaxel, epirubicin, cyclophosphamide	200	Recruiting	NA	NCT01929811
Breast	Phase II RCT	Clinical response rate	Letrozole 2.5mg PO daily, metformin 500mg BID PO daily x 1week; metformin 1000mg (am) & 500 mg (pm) PO daily x 1week; metformin 1000mg BID PO daily x 22 weeks	metformin, letrozole	208	Recruiting	NA	NCT01589367
Breast	Phase II RCT	To evaluate if using new chemotherapy drugs with standard neoadjuvant chemotherapy agents increases pCR.	AMG 479 (Ganitumab) 12 mg/kg IV every 2 weeks during 12 week cycle + metformin 850 mg PO every week during 12 weekly cycles post-randomization	metformin, ganitumab	800	Recruiting	NA	NCT01042379
Breast	Phase III RCT	Invasive disease-free survival	Metformin HCl PO BID (QD for first 4 weeks), for up to 5 years.	metformin monotherapy	3649	Active but not recruiting	NA	NCT01101438
Breast	Phase III RCT	Evaluating atypia after 12 and 24 months of metformin	Metformin HCl 850 mg PO BID for 24 months on arm 1, including 4 weeks escalation dosing of 850 mg PO QD. Patients initially randomized to the placebo arm (Arm 2) may crossover to the metformin arm (Arm 1) for months 13-24.	metformin monotherapy	400	Not yet recruiting	NA	NCT01905046
Chronic Lymphocytic Leukemia	Phase II	Time to treatment failure (assessed every 3 months)	Initial dose of metformin will be 500 mg PO QD for 1 week, escalated to 500 mg BID after 1 week, and finally to 1000 mg BID in week 3 if tolerated.	metformin monotherapy	53	Recruiting	NA	NCT01750567
Colorectal	Phase II	Disease Control Rate (assessed up to 1 year)	FOLFOX6+Metformin or FOFIRI + Metformin	metformin, FOLFOX6, FOFIRI	48	Recruiting	NA	NCT01926769
Endometrial	Phase II	CBR	Metformin 500 mg PO QD for days 1-4 of cycle 0 and then BID after that. Metformin to be taken for 7 - 10 days in cycle 0 before cycle 1 begins; letrozole 2.5 mg PO QD in a 28 day cycle; everolimus 10 mg PO QD in a 28 day cycle.	metformin, letrozole, everolimus	64	Recruiting	NA	NCT01797523
Endometrial	Phase II RCT	Pathological response rate (evaluated at 12 weeks)	Metformin 500 mg PO third daily and megestrol acetate 150 mg PO QD for 3 months	metformin, megestrol acetate	150	Recruiting	NA	NCT01968317
Endometrial	Phase II, III RCT	PFS (Phase II) (assessed up to 5 years)	Day 1- paclitaxel IV over 3 hours and carboplatin IV; metformin PO BID on days 1-21 (QD in cycle 1). Treatment repeats every 21 days for 6 cycles. Patients then receive maintenance therapy- metformin PO BID on days 1-21. Course repeated every 21 days.	metformin, paclitaxel, carboplatin	540	Recruiting	NA	NCT02065687
Glioblastoma Multiforme	Phase I	MTD and PFS assessed at 6 months	Temozolomide 150 mg/m^2^ PO on alternate weeks days 1-7, 15-21 of a 28 day cycle; metformin 1000 mg PO BID for 28 day cycle.	metformin, temozolomide	144	Recruiting	NA	NCT01430351
Lung	Phase II	PFS (assessed for up to 52 weeks)	Metformin 1000 mg PO BID added to standard of care platinum based chemotherapy	metformin, platinum based chemotherapy	60	Recruiting	NA	NCT02019979
Lung	Phase II RCT	PFS assessed at 1 year	Gefitinib + metformin; metformin starting at 500 mg TID PO. After 1 week, increase dose to 1000 mg as the 1st dose of the day and 500 mg as the 2^nd^ dose. After another week, increase to 1000 mg TID. Metformin treatment will be initiated 1 week before TKI therapy if possible.	metformin, gefitinib	168	Recruiting	NA	NCT01864681
Ovary	Phase I	Assess safety and tolerability of the treatment	Escalating doses of metformin will be added to carboplatin IV / paclitaxel IV chemotherapy for up to 6 cycles.	metformin, carboplatin, paclitaxel	20	Not yet recruiting	NA	NCT02312661
Ovarian, Fallopian Tube or Primary Peritoneal	Phase I	Success rates of the combination chemotherapy	Day 1- paclitaxel IV over 3 hours, carboplatin IV, metformin HCl BID on days 1-21; treatment repeated every 21 days for 6 cycles	metformin, paclitaxel, carboplatin	18	Not yet recruiting	NA	NCT02050009
Pancreas	Phase II	PFS at 12 months	Everolimus + octreotide LAR + metformin	metformin, everolimus, octreotide LAR	43	Recruiting	NA	NCT02294006
Pancreas	Phase II RCT	RFS after surgery (assessed every 2 months during treatment and every 3 months after completion, till 24 months)	Metformin 500 mg BID initially, if tolerated, dose to be increased to 1000 mg BID in the 2^nd^ week; gemcitabine 1000 mg/m^2^ (IV, 30 minutes) on days 1, 8, and 15 for 3 weeks, followed by 1 week without treatment, and also receive placebo (Arm I)/ metformin (Arm II) on days 1-28. Course repeated every 4 weeks for up to 6 cycles.	metformin, gemcitabine	300	Recruiting	NA	NCT02005419
Prostate	Phase I	DLT assessed at 28 days	Enzalutamide PO QD and metformin HCl PO BID. Courses repeat every 28 days	metformin, enzalutamide	24	Not yet recruiting	NA	NCT02339168
Solid tumors	Phase I	To evaluate DLT when metformin is added to a chemotherapy regimen that is well tolerated.	Stage 1- patients randomized to 2 arms- concurrent arm (metformin + chemo) and delayed arm (chemo alone). Stage 2- patients in delayed arm crossed over to concurrent arm; metformin dose- 500mg BID + chemotherapy	metformin, chemotherapy	100	Completed	Metformin plus chemotherapy had a lower rate of DLTs (6.1%) as compared to those who received only chemotherapy (7.8%); Patients showing DLT with metformin included the ones already known to have adverse effects with chemotherapy; Post-treatment with metformin, AMPK phosphorylation increased by 4-6 folds, 46% showed stable disease and 28% of the patients who had quantifiable tumor markers showed favorable changes.	NCT01442870 [49]
Uterus	Phase II RCT	pCR (assessed at 6 months)	Metformin 500mg BID PO + levonorgestrel 52mg IUD for 6 months	metformin, levonorgestrel	165	Recruiting	NA	NCT01686126

Abbreviations: RFS, recurrence free survival; PFS, Progression free survival; DLT, dose limiting toxicity; MTD, Maximum tolerable dose; pCR, Pathological complete response; RCT, Randomized control trial; CBR, Clinical benefit rate; IHC, immunohistochemistry; HOMA, Homeostasis model assessment; CI, Confidence interval; PO, taken by mouth; QD, once a day; BID, two times a day; TID, three times a day; IUD, Intra-uterine device

**Table 3:** Anti-tumor activity of metformin in metastatic tumors

Cancer type	Phase	Primary outcome/Conclusion	Dosing regimen	Combination	Enrollment No.	Status	Results	Clinical trial identifier/ Reference
Breast	Phase I	MTD of metformin when used with erlotinib	Initial metformin dose will be 850 mg BID and escalated to maximum FDA approved dose of 850 mg TID. DLT will be determined during the first 5 weeks of therapy; erlotinib 150 mg daily.	metformin, erlotinib	20	Recruiting	NA	NCT01650506
Breast	Phase II	PFS assessed at 2 months	Exemestane 25 mg PO QD, everolimus 10 mg PO daily and metformin 500 mg PO daily for 3 days. If DLT does not occur, metformin dose will be escalated by 500 mg PO every 3 days to reach target of 1000 mg PO BID	metformin, exemestane	40	Active but not recruiting	NA	NCT01627067
Breast	Phase II RCT	PFS (assessed up to 3 years)	Metformin 850 mg BID in addition to standard chemotherapy consisting anthracyclines, platinum, taxanes or capecitabine	metformin, standard chemotherapy (anthracycline, platinum, taxanes or capecitabine)	78	Recruiting	NA	NCT01310231
Breast	Phase II RCT	PFS (assessed up to 42 months)	Cycle 1- ONLY metformin from day 1-13, chemotherapy initiated from day 14; day 1-3- metformin 1000 mg QD, day 4- metformin 1000 mg BID; Myocet 60 mg/m2 IV on day 1, every 21 days; Cyclophosphamide 600 mg/m^2^ IV at day 1 every 21 days	metformin, myocet, cyclophosphamide	112	Recruiting	NA	NCT01885013
Colorectal	Phase II	Progression assessed at 12 weeks	Irinotecan 350mg/m² q21d + metformin up to 2500mg/day	metformin, irinotecan	41	Not yet recruiting	NA	NCT01930864
Colorectal	Phase II	Disease control rate (assessed every 8 weeks)	metformin 850mg PO BID plus 5FU 425mg/m2 plus leucovorin 50mg IV weekly	metformin, 5FU, leucovorin	50	Recruiting	NA	NCT01941953
Lung	Phase II RCT	PFS (assessed at 1 year) of metformin plus standard chemotherapy	Metformin started at 500 mg BID PO. Dose increased to 1000 mg as 1^st^ dose and 500 mg as 2^nd^ dose of the day after 1st week. After another week, dose will be 1000 mg BID. Metformin initiated 1 week before chemotherapy. Paclitaxel 200 mg/m² IV, day 1 of each cycle. Carboplatin at AUC= 6 mg/ml IV following paclitaxel, every 21 days. Bevacizumab 15 mg/kg every 21 days, after completion of chemotherapy, starting with cycle 1. After 4 cycles of induction chemotherapy, continue bevacizumab at 15 mg/kg every 21 days until disease progression.	metformin, paclitaxel, bevacizumab, carboplatin	60	Recruiting	NA	NCT01578551
Melanoma	Phase II	RR defined by the ratio between total number of patients with a complete response (CR) or partial response (PR).	2540 mg/day of metformin-base	metformin monotherapy	20	Unknown	NA	NCT01840007
Melanoma	Phase II RCT	RR	Dacarbazine 1000 mg/m^2^ once every 28 days with Metformin 850 mg BID	metformin, dacarbazine	96	Recruiting	NA	NCT02190838
Neuro-endocrine	Phase II	Disease free survival (assessed at 180 days)	Metformin 850mg BID	metformin monotherapy	30	Recruiting	NA	NCT02279758
Ovarian, fallopian tube, or primary peritoneal cancer	Phase II RCT	PFS	Chemotherapy options- 1. Paclitaxel IV over 2-3 hours + carboplatin IV on day 1; 2. Docetaxel IV over 1 hour + carboplatin IV on day 1; 3. Paclitaxel IV on days 1, 8, and 15 + carboplatin IV on day 1. Patients randomized to 1 of 2 treatment arms and will receive metformin PO BID with chemotherapy for 6 courses (21 days cycle).	metformin, paclitaxel, carboplatin, docetaxel	160	Recruiting	NA	NCT02122185
Pancreas	Phase I	To evaluate the toxicity, feasibility and compliance of chemotherapy regimen	Gemcitabine HCl and paclitaxel albumin-stabilized nanoparticle formulation IV on days 1, 8, 15. Patients also receive metformin PO BID from day (−)6 and dietary supplement PO BID from day (−)3. Cycles repeat every 28 days.	metformin, gemcitabine, paclitaxel	21	Not yet recruiting	NA	NCT02336087
Pancreas	Phase I, Phase II RCT	To evaluate feasibility and safety of metformin plus rapamycin versus rapamycin alone after stabilization of disease on chemotherapy.	Arm A- metformin 850mg PO BID on a 28 day cycle, Arm B- metformin 850mg PO BID plus rapamycin 4mg PO QD on a 28 day cycle.	metformin, rapamycin	22	Recruiting	NA	NCT02048384
Pancreas	Phase II	Radiological evidence of disease control	Paclitaxel 80 mg/m^2^ IV day 1, 8, 15; metformin 850mg PO TID	metformin, paclitaxel	41	Completed	6 patients (31.6%) showed stable disease; Median overall survival: 133 days; Median PFS: 43 days. 8 patients (42.1%) had treatment-related grade 3-4 toxicities with 6 cases (31.6%) requiring metformin dose reduction due to diarrhea; paclitaxel dose decreased in 1 case because of febrile neutropenia.	NCT01971034 [46]
Pancreas	Phase II	RR as determined by CT/MRI and PFS	Metformin PO BID days 1-14 and FOLFOX therapy comprising leucovorin calcium IV, fluorouracil IV, and oxaliplatin IV on day 1. Cycles repeated every 14 days.	metformin, FOLFOX (leucovorin calcium, fluorouracil, oxaliplatin)	43	Recruiting	NA	NCT01666730
Pancreas	Phase II RCT	PFS at 6 months	Cisplatin and epirubicin at 30 mg/m^2^ on days 1 and 15, gemcitabine at 800 mg/m^2^ on days 1 and 15, capecitabine at 1250 mg/m2 days 1-28, metformin at 2000 mg/day days 1-28	metformin, cisplatin, epirubicin, gemcitabine, capecitabine	82	Active but not recruiting	NA	NCT01167738
Pancreas	Phase II RCT	Survival after 6 months	Gemcitabine 1000 mg/m2 IV given weekly for 3 weeks, followed by 1 week without gemcitabine; erlotinib 100 mg daily; metformin 500 mg BID, if tolerated, dose will be increased to 1000 mg BID in 2^nd^ week.	metformin, gemcitabine, erlotinib	120	Completed	6 month survival rate for metformin arm was 55% (95% CI: 42-68) and for placebo arm was 66% (95%CI: 54-78, p= 0.23); Median overall survival was 6.8 months (95%CI: 5.3-8.3) and PFS was 4.4 months (95%CI: 2.0-6.7) in metformin arm and for placebo arm were 7.6 (95%CI: 6.3-9.0) and 5.4 (95%CI: 4.7-6.1) months respectively; Metformin well tolerated, when compared to placebo showed no significant variation recorded in grade ≥3 toxicity.	NCT01210911 [47]
Prostate	Phase II	PFS (assessed at 12 weeks)	Metformin lifelong follow-up at 1000 mg BID daily until progression, unacceptable toxicity or refusal of therapy	metformin monotherapy	44	Active but not recruiting	NA	NCT01243385
Prostate	Phase II	PFS (assessed at 12 weeks)	Addition of metformin 1000 mg PO BID to abiraterone in case of PSA progression	metformin, abiraterone	25	Recruiting	NA	NCT01677897
Prostate	Phase II RCT	PSA response	Docetaxel 75 mg/m^2^, metformin 850 mg PO BID daily	metformin, docetaxel	100	Recruiting	NA	NCT01796028
Advanced solid tumors	Phase I	MTD of metformin when used in combination with temsirolimus	Metformin 500 mg PO QD/BID/TID daily on days 1-28 and temsirolimus 15-25 mg IV weekly	metformin, temsirolimus	11	Completed	- 3/3 patients in 1^st^ cohort had DLTs including grade 4 pneumonitis, grade 3 fatigue and grade 3 thrombocytopenia - 2/8 patients had DLT in 2^nd^ cohort: grade 4 dyspnea and grade 3 thrombocytopenia	NCT00659568 [50]
Advanced cancers/ metastatic cancers	Phase I	MTD of temsirolimus and metformin	Temsirolimus 25 mg IV weekly; metformin titrated over 3 weeks at 500 mg PO daily. 4 weeks of treatment constitute 1 cycle. Cycle 1 to be 6 weeks to allow metformin titration.	metformin, temsirolimus	104	Recruiting	NA	NCT01529593
Advanced cancers/ metastatic cancers	Phase I	MTD of lapatinib with sirolimus or metformin	Lapatinib starting dose 500 mg PO daily for 21 day cycle, metformin starting dose 1000 mg PO daily.	metformin, lapatinib	106	Active but not recruiting	NA	NCT01087983

Abbreviations: RR, recurrence rate; PFS, Progression free survival; PSA, Prostate specific antigen; MTD, Maximum tolerable dose; DLT, Dose limiting toxicity; pCR, Pathological complete response; RCT, Randomized control trial; CBR, Clinical benefit rate; IHC, Immunohistochemistry; CI, Confidence interval; PO, taken by mouth; QD, once a day; BID, two times a day; TID, three times a day

**Table 4:** Antitumor activity of metformin in combination with radiotherapy

Cancer type	Phase	Primary outcome/Conclusion	Dosing regimen	Combination	Enrollment No.	Status	Results	Clinical trial identifier/Reference
Brain	Phase I	Number of participants that had adverse events and the number that completed the trial (assessed at 8 weeks)	Partial brain re-irradiation to a dose of 30-35Gy over 2 weeks (10 fractions) combined with low carbohydrate diet and/or metformin	metformin, partial brain re-irradiation	18	Not yet recruiting	NA	NCT02149459
Head and neck locally advanced squamous cell carcinoma	Phase I	MTD of metformin when used with cisplatin plus radiation	metformin administered in escalating doses of 2000mg, 2550 mg and 3000mg PO starting 1 week prior to the initiation of chemoradiation and ending on the last day of chemo or radiation. Cisplatin 100mg/m2 on days 1, 22, and 43, Radiation 70 Gy in 2 Gy once daily fractions of 35 fractions.	metformin, chemo-radiation (with cisplatin)	30	Not yet recruiting	NA	NCT02325401
Lung	Phase II RCT	Comparing response to metformin versus placebo	Metformin for 3 weeks prior to SBRT and for 1 week during SBRT. Metformin dose- 2000 mg/day PO (500 mg AM, 1000 mg noon, 500 mg PM). Metformin started at 1000 mg daily in divided doses for 1 week. SBRT delivered per standard of care.	metformin, SBRT	70	Recruiting	NA	NCT02285855
Lung	Phase II RCT	PFS (assessed for up to 12 months)	Metformin 500 mg BID PO for week 1, 1500 mg/day in week 2 and 2000 mg/day at week 3 and then for a period of 12 months. Cisplatin-based chemotherapy with standard radiotherapy of 60-63 Gy for 6 weeks.	metformin, cisplatin-based chemotherapy, standard radiotherapy	94	Recruiting	NA	NCT02115464
Lung	Phase II RCT	PFS (assessed for up to 5 years)	Metformin PO BID or TID x14 days. Day 15 onwards patients receive paclitaxel IV and carboplatin IV on days 1, 8, 15, 22, 29, 36 and undergo radiation therapy (3D-CRT or IMRT) QD 5 days/week for 6 weeks and receive metformin BID/TID for 6 weeks. From 28-42 days after radiotherapy, patients receive consolidation chemotherapy with paclitaxel IV and carboplatin IV on days 1 and 22 and metformin PO BID/TID for 10 weeks. Consolidation chemotherapy repeated every 3 weeks for 2 courses.	metformin, paclitaxel, carboplatin, radiotherapy (3D-CRT or IMRT), consolidation chemotherapy with paclitaxel and carboplatin	168	Recruiting	NA	NCT02186847
Pancreas	NA	DLT (assessed up to 21 days after therapy)	Metformin PO or BID on days (−)11 to (−)1. Patients then undergo stereotactic radiosurgery 5 days/week and receive concurrent metformin PO BID for 5 weeks. Patients undergo laparotomy in week 6 (or weeks 5-7). Systemic therapy continues as soon as it is considered feasible by treating physicians.	metformin, stereotactic radiosurgery	20	Not yet recruiting	NA	NCT02153450

Abbreviations: PFS, Progression free survival; DLT, dose limiting toxicity; MTD, Maximum tolerable dose; RCT, Randomized control trial; SBRT, Stereotactic body radiation therapy; 3D-CRT, 3D Conformal radiotherapy; IMRT, Intensity modulated radiation therapy; PO, taken by mouth; QD, once a day; BID, two times a day; TID, three times a day

A considerable amount of focus has been laid on investigating metformin as a potential anti-cancer agent for cases of breast cancer. Eleven trials (20% of all ongoing trials using metformin as an anti-cancer agent; Tables 1, 2, 3) are focused on evaluating metformin as a treatment for breast cancer. Of these, two trials are using metformin as monotherapy. There are 9 trials using metformin in combination with other anti-cancer agents. These include capecitabine, cyclophosphamide, docetaxel, doxorubicin, erlotinib, epirubicin, exemestane, ganitumab, letrozole, sirolimus and temsirolimus. One trial is exploring the use of metformin plus atorvastatin combination as a possible treatment for breast cancer. Two trials using metformin combination therapy are also evaluating pathological complete response as a primary endpoint. Apart from the ongoing trials, data obtained from 5 completed trials (all using metformin monotherapy in a pre-surgical window of opportunity trial design) has facilitated a better understanding regarding the effects of metformin in breast cancer. In addition to survival outcomes, several surrogate markers are also being employed to study the effects of metformin on breast cancer cell population. These include Ki67, S6K, 4E-BP-1, AMPK and effects on AMPK/mTOR pathway.

A phase 2 single arm window of opportunity trial of 39 breast cancer cases [37] showed significant reduction in Ki67 (36.5 to 33.5 %, *p* = 0.016) and an increase in TUNEL staining (0.56 to 1.05, *p* = 0.004) along with significant fall in HOMA (homeostatic model assessment, used for determining the status of insulin resistance) [38]. A recently published randomized control trial (RCT) also reported a decrease in Ki67 staining (mean = 3.4%, *p* = 0.027). Additionally, it noted an increase in mean AMPK score, fall in pAKT score and reduced caspase-3 staining in patient samples with the use of metformin when compared to placebo [39]. However, other trials have had conflicting outcomes. A phase II RCT with 200 participants recorded no significant changes in Ki67 on comparing metformin and placebo arms. But, interestingly, the cases with HOMA ≤ 2.8 showed a non-significant increase of Ki67 by 11.1% (95% Confidence interval (CI): −0.6% to 24.2%) and those with HOMA > 2.8 (implying a higher probability of insulin resistance) showed a non-significant mean proportional decrease in Ki67 by 10.5% (95% CI: −26.1% to 8.4%) [40]. Another phase II trial (non-randomized) examined effects of metformin in overweight/obese patients with stage 0-III breast cancer. Though noting a correlation of Ki67 with tumor growth, the calculated ln (Ki67) showed no significant changes when comparing metformin to placebo [41]. A different study with 200 participants randomized to metformin or placebo did not document any major difference in Ki67 and TUNEL levels (used for assessing cellular apoptosis) between the two arms. The study did note that TUNEL levels were higher in women without insulin resistance (metformin: +4%, interquartile range, IQR: 2-14, placebo: +2%, IQR: 0-7) as compared to those who had insulin resistance (metformin +2%, IQR: 0-6, placebo +5%, IQR: 0-15) [42]. The survival benefit with the use of metformin in breast cancer is being evaluated in 3 clinical trials, however there is no data presently available (NCT01627067, NCT0131023, NCT01885013).

Metformin is presently being evaluated as an anti-cancer agent for endometrial cancer as well. There are 6 ongoing trials (10.9% of all ongoing trials using metformin as an anti-cancer agent; Tables 1, 2), with 3 each for monotherapy and combination chemotherapy. The drugs that are currently being assessed in combination with metformin for treatment of endometrial cancer are carboplatin, everolimus, letrozole, paclitaxel, and megestrol acetate. One trial is assessing the role of metformin as a maintenance therapy. In conjunction with clinical response, the effect of metformin on endometrial cancer is being studied through a wide variety of markers including Ki67, pS6, Akt, pAMPK, ERK1/2, histone H3, telomerase, topoisomerase IIα and p27. The effect of using metformin on expression of estrogen (ER) and progesterone (PR) receptors in cancer tissue of endometrial origin is also being investigated. Three completed trials, all pre-surgical window of opportunity trials using metformin monotherapy, have shown a significant decrease in Ki67 staining [43–45] and in pS6 staining [44, 45]. One trial also reported a reduction in topoisomerase IIα and ERK 1 / 2, along with significant elevation in pAMPK and p27 [45]. A different trial reported a decrease in tumor cell proliferation by 11.75% and a decrease in expression of ER with the use of metformin. PR expression, however, was not affected [43]. Thus far, no trial has provided any data on survival benefit with the use of metformin in endometrial cancer, though one trial is ongoing (NCT02065687).

Metformin is being assessed in combination with various anti-cancer agents for the treatment of pancreatic cancer. Presently, there are 7 ongoing and 2 completed trials (Tables 2, 3, 4). With the exception of one trial where the treatment regimen involves using metformin together with stereotactic radiosurgery (Table 4), all others are using metformin in combination with different anti-cancer agents. These include cisplatin, capecitabine, epirubicin, erlotinib, everolimus, gemcitabine, octreotide, paclitaxel, rapamycin, and FOLFOX (fluorouracil, oxaliplatin, leucovorin). The effects of metformin are being assessed mostly through clinical outcomes including progression free survival (PFS), recurrence free survival (RFS) and toxicity due to chemotherapy combination. The results from a phase II non-randomized trial showed that the combination of metformin plus paclitaxel was not well tolerated, with 42.1% patients experiencing grade 3-4 toxicities. A total of 31.6% cases had to undergo metformin dose reduction secondary to development of diarrhea, while one case experienced febrile neutropenia which was attributed to paclitaxel. This trial reported a median overall survival (OS) of 133 days and median PFS as 43 days, but could not meet the disease control rate endpoint [46]. Another trial, consisting of 120 participants randomized to metformin or placebo arm, noted that although the combination of metformin, gemcitabine and erlotinib was well tolerated, the 6 month survival rate was 55% in metformin arm and 66% in placebo arm. Also, no significant difference was observed in PFS and median OS between metformin users and non-users [47].

Five clinical trials (Tables 1, 2, 3) are presently working to evaluate if metformin may be of value in the treatment of prostate cancer. These include two trials that are using metformin as monotherapy and three in combination with different agents: abiraterone (NCT01677897), docetaxel (NCT01796028), and enzalutamide (NCT02339168). Data made available from one trial, a single arm window of opportunity study, showed a significant reduction in Ki67 index and 4E-BP-1 staining with no changes in pAMPK. Three of 24 patients developed grade 3-4 toxicities, indicating that the treatment was overall well tolerated [48]. The effect of metformin therapy on PFS for prostate cancer is being assessed in two trials (NCT01433913, NCT02339168), with one other trial evaluating PSA response (NCT01796028). However, presently there is no data available on survival benefit.

There is one phase II trial of metformin use in non-small cell lung cancer (NSCLC) combined with stereotactic body radiotherapy that is currently recruiting patients (NCT02285855).

There are two completed trials (Table 2, 3) on multi-histology solid tumors assessing the dose limiting toxicity (DLT) of various treatment regimens that include metformin. One of the trials used metformin in combination with 26 chemotherapy regimens for 17 tumor types on a total of 100 participants. The study was divided in two stages. In stage one, participants were randomized to receive metformin or placebo with chemotherapy. In stage two, participants of delayed arm would be crossed over to receive metformin with chemotherapy. Results showed that 46% of participants documented stable disease. In the sub-set of patients having quantifiable tumor markers, 28% exhibited favorable changes. The participants receiving metformin together with chemotherapy showed a lower rate of DLT (6.1% in stage one of concurrent arm, including grade 3 anemia, decrease in albumin and elevation in ALT) compared to those who received just the chemotherapy (7.8% in stage one of delayed arm [including grade 3 syncope, dehydration and elevation of bilirubin] and 3.8 % in stage two [including dehydration, vomiting and proteinuria]). The participants reporting DLTs in stage two of delayed arm were known cases of adverse events with chemotherapy [49]. The other trial used metformin and temsirolimus combination in 11 patients. It reported that 100% of participants had grade 1 toxicity and 82% experienced grade 2 toxicity, with DLT being reported in all 3 patients of the first cohort (grade 4 pneumonitis, grade 3 fatigue and grade 3 thrombocytopenia). In the second cohort, the dose of temsirolimus and metformin was reduced and DLT was observed in only two of eight cases (grade 4 dyspnea and grade 3 thrombocytopenia). After 2 months of treatment, 5 patients had stable disease, 1 case had partial response and 2 showed progression [50].

## DISCUSSION

There is great excitement surrounding metformin as a potential anti-cancer agent. Epidemiological data has associated the use of metformin with a decrease in the risk of developing cancer and a reduced cancer related mortality. The information that has been gathered from preclinical studies has provided encouraging evidence for anticancer mechanisms of metformin. It has been suggested that metformin may well be used as a radiation sensitizer or an immunotherapy drug, in addition to a direct anti-proliferative agent for the treatment of cancer.

The anticancer mechanism of metformin has been extensively studied and attributed to mTOR inhibition. More recent data has revealed an immunomodulatory effect on cancer cells. Pre-clinical data has demonstrated that metformin can inhibit apoptosis of CD8+ TILs. In addition, it also increases the effector memory T cell population through phenotype switching of CD8+ TILs, thus enhancing the immune response against tumor cells [26]. The use of metformin with an experimental cancer vaccine *(LmOVA)* showed an increase in the number of CD8+ memory T cells that conferred immunity to cancer [27]. The recent breakthrough developments in immunotherapy for patients with advanced melanoma, triple negative breast cancer, and non-small cell lung cancer with check point blockade monoclonal antibodies (anti-PD-1/L1) have generated excitement in the oncology community [51–53]. Immunomodulatory properties of metformin have yet to be studied in combination with other forms of immunotherapy, in particular with check point blockade monoclonal antibodies. Further investigation into a possible synergistic effect is warranted.

The preliminary results from clinical trials assessing metformin as an anti-cancer agent have shown that metformin can significantly impact markers of tumor proliferation. A total of 19 ongoing and completed trials (Table 1) are using various surrogate markers to assess pro-apoptotic effects of metformin on cancer cells. Although majority of these trials are being performed on breast and endometrial cancer cases, a limited number of trials are also evaluating tumors of head & neck, prostate, bladder, lung, kidney and lymphoma.

Pre-surgical window of opportunity trials in endometrial and breast cancer showed that tumor markers such as Ki67 and TUNEL (indicative of changes in cell proliferation and apoptosis respectively) exhibited favorable anti-tumor effect. Two trials demonstrated favorable changes in Ki67 and TUNEL in a subset of women without insulin resistance as compared to those with insulin resistance. Although the findings were not statistically significant, the direction of change suggests an intriguing hypothesis. The use of metformin in patients without insulin resistance may offer more benefit as compared to those with insulin resistance.

Diabetes is known to be associated with insulin resistance [54] and an impaired immune response against various pathogens [55, 56]. The immune system responds to the proliferating tumor cells by increasing production of tumor specific lymphocytes which check tumor growth by various mechanisms [57]. Evidence from preclinical trials has described that metformin, at least in part, exerts an anti-cancer effect by inhibiting immune exhaustion of CD 8+ TILs [26], thus amplifying the existing immune action against cancer cells. Therefore, it may be hypothesized that the patients with insulin resistance have a compromised immune system, which consequently results in a sub-optimal anti-cancer effect of metformin. It might be rational to stratify outcomes according to the insulin resistance status of participants in future clinical trials in order to better appreciate the anti-cancer activity of metformin.

Fourteen ongoing trials (Tables 1, 2, 3, 4) are presently amassing evidence to ascertain if a survival benefit is associated with the use of metformin in various malignancies. These include tumors of the breast, pancreas, lung, endometrial, brain, prostate and gynecological cancers. Thus far, there are results available from two clinical trials on metastatic pancreatic cancer, neither of which had favorable outcomes. From this data, it can be speculated that metformin may not be viable option for the treatment of advanced pancreatic cancer. The upcoming clinical trials may need to shift focus towards treating earlier stages of pancreatic cancer or using a different combination of agents with metformin to have better outcomes in advanced disease. Additional data on survival indices from multiple ongoing trials will be pivotal to draw a better conclusion.

One concern with the clinical utility of metformin is its side effect profile, particularly in combination with cytotoxic chemotherapy. Metformin is well known for causing GI upset, sometimes limiting patient compliance due to discomfort. Clinical trials have revealed a low incidence of DLTs with metformin in combination with a wide variety of chemotherapy regimens [48]. With this data, clinicians can be reassured that metformin will most likely be a tolerable addition to a chemotherapy regimen, and should not limit its practical utility.

## CONCLUSIONS

A strong base of epidemiological and pre-clinical data has prompted attempts to probe the anti-cancer effects of metformin through clinical trials. Metformin has been shown to have a favorable effect on markers of tumor proliferation but it remains to be seen if that translates to benefit in survival rates. It is prudent to find better histology and the appropriate stage of tumors for utilizing metformin therapy. The potential use of metformin as an immunotherapy agent needs to be substantiated with further evidence to ascertain possible benefits in future.
